# Zero-strain reductive intercalation in a molecular framework†
†Electronic supplementary information (ESI) available: Synthesis, experimental methods, and sample characterisation; X-ray powder diffraction refinement details. See DOI: 10.1039/c4ce02364a



**DOI:** 10.1039/c4ce02364a

**Published:** 2015-01-16

**Authors:** Joshua A. Hill, Andrew B. Cairns, Jared J. K. Lim, Simon J. Cassidy, Simon J. Clarke, Andrew L. Goodwin

**Affiliations:** a Department of Chemistry , University of Oxford , Inorganic Chemistry Laboratory , South Parks Road , Oxford , OX1 3QR , UK . Email: andrew.goodwin@chem.ox.ac.uk ; Fax: +44 (0)1865 274690 ; Tel: +44 (0)1865 272137

## Abstract

Reductive intercalation of potassium within the molecular framework Ag_3_[Fe(CN)_6_] gives rise to a volume strain that is an order of magnitude smaller than is typical for common ion-storage materials. We suggest that framework flexibility might be exploited as a general strategy for reducing cycling strain in battery and ion-storage materials.

## Abstract 2

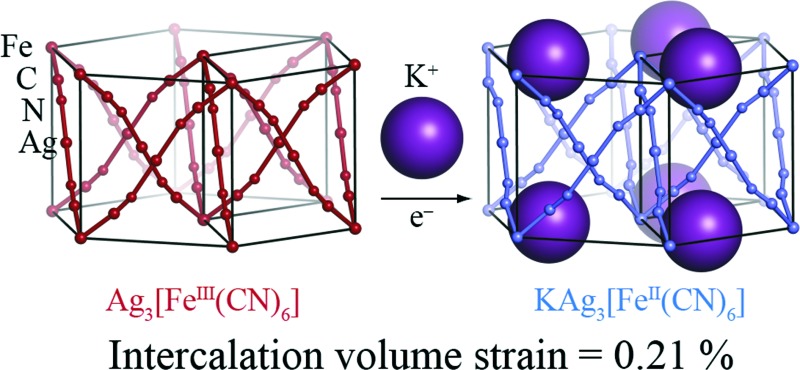
Intercalation of potassium into the molecular framework silver hexacyanoferrate occurs with remarkably small volume strain.

One of the central considerations in the development of ion-storage materials is the effect of mass transport on bulk physical properties of the host material.^1^ The existence of large structural changes on charge or discharge increases the propensity for dislodging, cracking and degradation—all of which reduce battery life and long-term storage capacity.^2–4^ Upon intercalation most ion-storage media undergo molar volume expansions of between 1 and 10%:^4^ a response equivalent to a temperature increase of *ca.* 2000 K.^5^ The practical consequences of such a large ‘electrochemical shock’ are so severe that much of the field has focussed on developing inventive work-arounds to accommodate volume strain. So, for example, one strategy is that of nanofabrication: by preparing battery materials in the form of nanowires or thin-films it is possible to sustain much larger volume strains, albeit at the expense of storage density.^6,7^

Ion-storage media that can accommodate Na^+^ or K^+^ are increasingly sought-after as battery electrode materials.^8,9^ These earth-abundant elements could be used in place of lithium for large, static batteries where energy density is much less important compared to mobile applications. One of the challenges in developing electrodes for such batteries is accommodating these much larger ions without drastic volume changes.

We were intrigued by the possibility of exploiting structural flexibility in molecular framework materials as a means of engineering systems with much-reduced *intrinsic* volume strains. Our particular focus on molecular frameworks has been motivated by the increasingly-apparent predisposition of such materials to anomalous temperature- and pressure-dependent strain behaviour. For example, negative thermal expansion (NTE, volume reduction on heating) is rarely observed in conventional engineering materials^10^ but is not at all uncommon amongst molecular frameworks;^11–13^ moreover this NTE effect can be tensioned against the more usual positive thermal expansion contributions of other structural motifs to produce materials with near-zero coefficients of thermal expansion.^14^ Negative and near-zero compressibilities are also achievable in similar systems.^15–17^ Such unconventional mechanical responses are rationalised in terms of geometric flexibility: the ease with which molecular frameworks can vary their lattice geometries allows behaviour not observed in conventional materials.^18^ Given that thermal and pressure-induced strains can be ameliorated by framework flexibility, our hope was that electrochemical strain might also be fundamentally reduced in suitably-chosen molecular frameworks.

Perhaps the key difficulty faced in developing molecular-framework-based ion-storage materials is that redox chemistry is surprisingly rare for these systems.^19^ The few molecular frameworks for which bulk insertion has actually been demonstrated include MIL-53(Fe),^20,21^ lithium isopropoxide-modified magnesium 2,5-dioxido-1,4-benzenedicarboxylate,^22^ Zn_0.5_Co_0.5_(HCOO)_3_,^23^ and the Prussian Blue analogue K_2_Cu[Fe(CN)_6_].^24^ Unfortunately, for each of these systems electrochemical strain is not especially reduced relative to “traditional” ion-storage materials such as Li_*x*_CoO_2_, and in some cases is actually much larger.^25^ Encouraged both by a voltammetric study of the [Fe^II/III^(CN)_6_]^4–/3–^ couple^26^ and by the discovery of extreme flexibility in silver(i)/gold(i) hexacyanometallates,^27^ we have chosen to focus on reductive intercalation in silver(i) hexacyanoferrate(iii), Ag_3_[Fe(CN)_6_] [Fig. 1(a)]. Our X-ray powder diffraction measurements, which are discussed below, reveal that the intrinsic volume strain associated with potassium intercalation in this system is an order of magnitude smaller than for typical ion-storage materials. This ranks Ag_3_[Fe(CN)_6_] amongst the few known “zero-strain” insertion compounds (Li_4_Ti_5_O_12_,^28^ 2,6-napth(COOLi)_2_,^29^ and Na_0.84_Ni[Fe(CN)_6_]_0.71_ (ref. 30)) in spite of the relatively large ionic radius of potassium [*r*(K^+^) = 1.52 Å *cf. r*(Li^+^) = 0.90 Å and *r*(Na^+^) = 1.16 Å].^31^

**Fig. 1:**
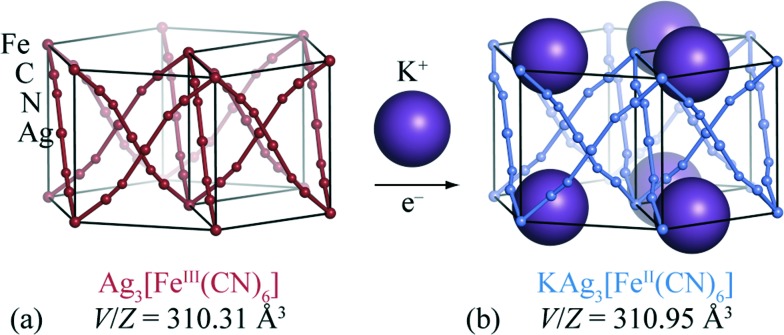
(a) The trigonal structure of Ag_3_[Fe(CN)_6_] consists of three interpenetrating α-Po cubic nets. (b) Reductive intercalation of K^+^ ions within this structure gives rise to the structurally-related phase KAg_3_[Fe(CN)_6_]. The K^+^ ions occupy one-dimensional channels which run parallel to the *c* crystallographic axis (vertical).

The crystal structure of Ag_3_[Fe(CN)_6_] has been reported previously:^26,27^ octahedral Fe^3+^ centres are connected *via* almost-linear dicyanoargentate ions to form three interpenetrating cubic nets [Fig. 1(a)]. The structure has *P*31*m* crystal symmetry, and includes a set of small vacant channels that run parallel to the trigonal *c* crystal axis. X-ray powder diffraction patterns for KAg_3_[Fe(CN)_6_] have twice been reported,^26,32^ but to the best of our knowledge its crystal structure has not yet been determined. In contrast, the structure of the Mn analogue KAg_3_[Mn(CN)_6_] is known: it is closely related to that of Ag_3_[Fe(CN)_6_] except with one half of the channels occupied by K^+^ cations, lowering the crystal symmetry to *P*312.^16^ This close structural similarity suggests that cation insertion within Ag_3_[Fe(CN)_6_] framework might be possible *via* straightforward inclusion of K^+^ ions within these same channels.

A sample of Ag_3_[Fe(CN)_6_], prepared as described previously,^27^ was reacted at 0 °C with stoichiometric quantities of potassium naphthalenide, using dried THF as solvent and working under a dinitrogen atmosphere on a Schlenk line. The solid product obtained is air-stable and exhibits a qualitatively similar X-ray powder diffraction pattern to the parent Ag_3_[Fe(CN)_6_] (as noted in ref. 26) [Fig. 2(a)]. On close inspection, small but meaningful shifts in the Bragg reflection positions and intensities are evident; we note that the difference between the diffraction patterns attributed to Ag_3_[Fe(CN)_6_] and KAg_3_[Fe(CN)_6_] in ref. 26 map quantitatively onto to those observed here. Rietveld refinement of our powder diffraction data (performed using TOPAS^46^) using a structural model for KAg_3_[Fe(CN)_6_] based on published coordinates for KAg_3_[Mn(CN)_6_] (ref. 16) gave an acceptable fit [Fig. 2(a)] and a physically-sensible set of refined parameters (see ESI† for full details). Our refinements indicated a small but significant variation in the lattice parameters (*a* = 7.0279(5) Å, *c* = 7.2546(5) Å for the vacant framework and *a* = 7.06984(23) Å, *c* = 7.1836(3) Å after intercalation), together with a K-site occupancy of 0.920(13). Infrared absorption spectra measured for starting material and product also reveal the redshift in CN stretching frequencies characteristic of Fe^III^/Fe^II^ reduction [Fig. 2(b)].^33,34^ The presence of a small quantity of remnant Fe^III^ is suggested by a weak feature in the infrared absorption spectrum near 2170 cm^–1^ [Fig. 2(b)] and will likely include contributions both from intercalated product (since the K-site occupancy is slightly less than unity) and a small fraction of unreacted Ag_3_[Fe(CN)_6_] also evident in the diffraction data (see ESI†). Nevertheless the dominant product is consistent with the formulation K_0.92_Ag_3_[Fe(CN)_6_] (we use KAg_3_[Fe(CN)_6_] hereafter for convenience) and with a structural model in which K^+^ ions are incorporated within one half of the initially-vacant framework channels.

**Fig. 2:**
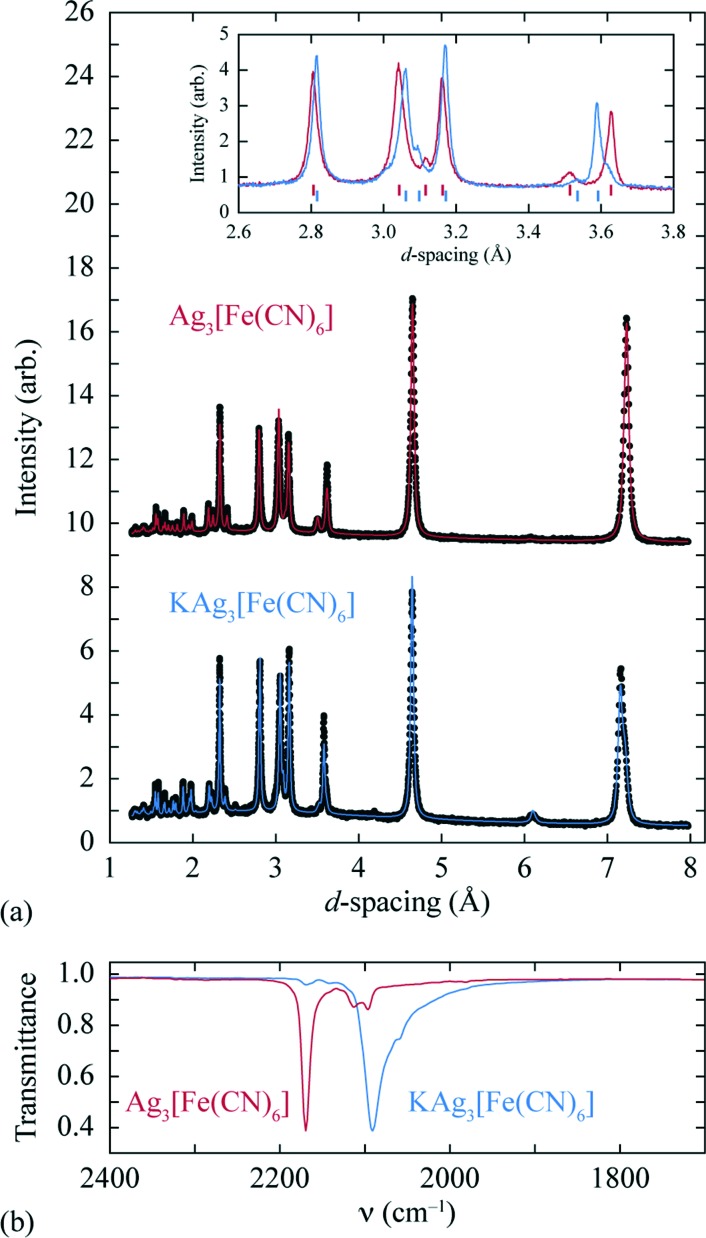
(a) Powder X-ray diffraction patterns and Rietveld fits for the empty (top, Ag_3_[Fe(CN)_6_]) and intercalated (bottom, KAg_3_[Fe(CN)_6_]) frameworks. A representative section of the diffraction pattern, highlighting the small shifts in peak positions and intensities, is shown in the inset. (b) The change in CN stretching frequencies on intercalation is as expected for Fe^III^/Fe^II^ reduction.^33,34^

The volume strain associated with potassium ion intercalation is determined straightforwardly by the variation in lattice constants. We find *V* = 310.31(5) Å^3^ for the vacant framework and *V* = 310.95(3) Å^3^ after intercalation, corresponding to a volume strain Δ*V*/*V* = +0.206(18)%. Table 1 places this value in the context of volume strains measured for a range of topical and well-known ion storage compounds; these data are represented graphically in Fig. 3, where they are distributed according to stored charge density. While this density is (understandably) lower for KAg_3_[Fe(CN)_6_] than most Li- and Na-based systems, the volume strain of intercalation is essentially identical to that of the “zero-strain” Li-ion storage material Li_2.33_Ti_1.67_O_4_.^37^

**Table 1:** Intercalation volume strains for some topical and canonical ion-storage compounds. The intercalated ion is indicated in bold

Compound	Normalised intercalation volume change (Å^3^/e^–^)	Stored charge density (×10^–3^ e^–^/Å^3^)	Intercalation strain, *ε*_V_ = |Δ*V*/*V*| (%)	Ref.
**Mg**FeSiO_4_	0.75	46.86	3.53	35
**Li**CoO_2_	0.86	9.87	0.85	25
**Li**FePO_4_	–4.80	14.71	7.06	36
**Li**_2.33_Ti_1.67_O_4_	0.15	13.69	0.20	37
**Li**_2_[2,6-napth(COOLi)_2_]	–0.39	8.55	0.33	29
**Li**C_6_	1.12	18.90	2.12	38
**Na**_3_Mn_2_(CN)_6_	–1.39	9.78	1.36	39
**Na**_1.5_VP_4.8_F_0.7_	–3.23	9.50	3.07	40
**Na**_2_FePO_4_F	3.99	9.39	3.75	41
**Na**_0.84_Ni[Fe(CN)_6_]_0.71_	–1.95	3.02	0.59	30
**Na**_2_FeP_2_O_7_	–6.32	5.34	3.37	42
**Na**_3_V_2_(PO_4_)_3_	–10.08	9.08	9.15	43, 44
**Na**_2_Mn_2_(CN)_6_	8.50	3.24	2.75	39
**K**_2_Cu[Fe(CN)_6_]	–9.55	2.84	2.71	24
**K**_0.92_Ag_3_Fe(CN)_6_	–0.70	2.96	0.21	This work
**KC**_8_	–41.24	14.13	58.28	45

**Fig. 3:**
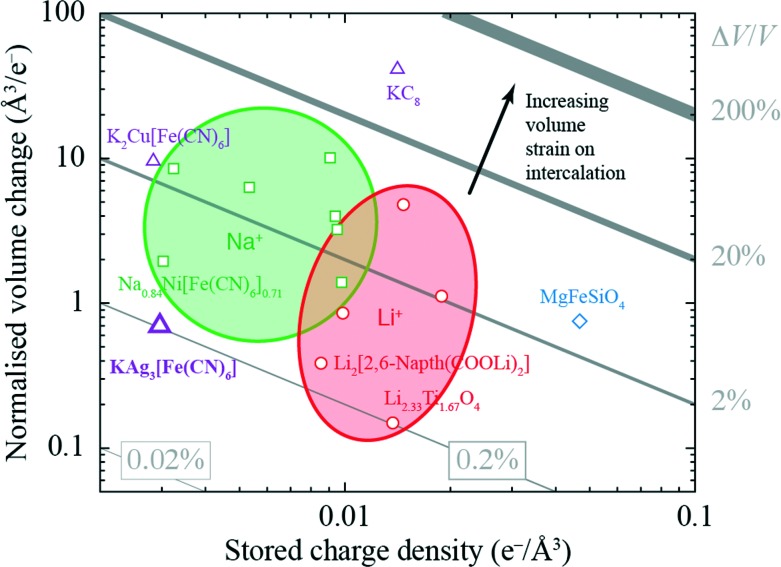
Ashby-type diagram for the ion-storage compounds of Table 1, with data points coloured by intercalant (Mg – blue, Li – red, Na – green, K – purple). The diagonal lines connect points of constant intercalation volume strain *ε*_V_ = Δ*V*/*V*. Different applications place different demands on stored charge density (*e.g.* portability *vs.* cost), yet in all cases it is desirable to minimise intercalation strain.

The small intercalation strain of KAg_3_[Fe(CN)_6_] is explained by the compensating changes in lattice parameters during K^+^ insertion. On intercalation, the framework expands modestly along the *a* and *b* crystal axes in order to widen the channels and accommodate the extra-framework cations. The crucial point is that expansion of the framework in one set of directions results in a contraction along the *c* axis—the framework behaves much like a three-dimensional “wine-rack” by simply flexing whilst maintaining its basic framework dimensions. So the Fe···Fe separation across connected Fe–CN–Ag–NC–Fe linkages (
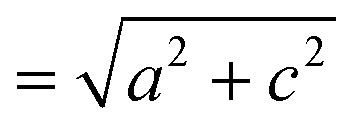
) changes by just 0.2% on intercalation, whereas the angle between adjacent linkages (given by 
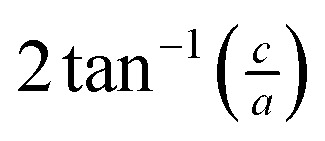
) changes by 1.0% and hence accounts for most of the (small) strain observed. This behaviour contrasts with the responses observed in ion-storage materials based on layered or dense framework structures. In the former instance (*e.g.* TiS_2_), intercalation simply results in increasing interlayer separation; and in the latter (*e.g.* LiFePO_4_), the whole lattice expands to accommodate the intercalant. For both types of system the volume strain is inherently large and positive because each of the linear strains is positive. The key advantage offered by molecular frameworks as ion-storage media is that their mechanical responses—whether to variations in temperature or pressure, or to guest inclusion—can be tailored to ensure an expansion in one or more directions is counteracted by a contraction in other directions.^13,16,47^ Whereas the linear strains induced by changes in temperature and/or pressure can be anomalously large, here we find that the strains can be much smaller than those observed in conventional systems; for example, the linear strain anisotropy Δ*ε* = *ε*_max_ – *ε*_min_ in the “zero strain” material Li_2_[2,6-napth(COOLi)_2_] is 22.70%, but is only 1.58% for KAg_3_[Fe(CN)_6_].^29^

In order for this “zero-strain” property of Ag_3_[Fe(CN)_6_] to find practical application, it remains to demonstrate the reversibility of the ion storage mechanism we explore here. Similarly, the use of other alkali metals (*e.g.* Li) and fine-tuning of the redox potentials by substitution at the Fe site are avenues for future work. The key result of this preliminary investigation is to demonstrate how the crucially important problem of intercalation strain might, in principle, be overcome by exploiting geometric flexibility whether such an approach is based on hexacyanometallate chemistry or otherwise.
