# Supplementary material for: Zero-strain reductive intercalation in a molecular framework
Source: CrystEngComm. 2015 Jan 16;17(15):2925–8. doi: 10.1039/c4ce02364a (PMC4396389; doi:10.1039/c4ce02364a)
Supplement: Supplementary file 1 [file CE-017-C4CE02364A-s001.pdf]

Electronic Supplementary Material (ESI) for CrystEngComm.
This journal is © The Royal Society of Chemistry 2015

Zero-strain reductive intercalation in a molecular framework

SUPPLEMENTARY INFORMATION

Joshua A. Hill,[1] Andrew B. Cairns,[1] Jared Lim,[1] Simon J. Cassidy,[1]
Simon J. Clarke,[1] and Andrew L. Goodwin[1*]

[1]Department of Chemistry, University of Oxford, Inorganic Chemistry Laboratory,
South Parks Road, Oxford OX1 3QR, U.K.

*To whom correspondence should be addressed; E-mail: andrew.goodwin@chem.ox.ac.uk.

Submitted to CrystEngComm

1 Materials and Methods

Synthesis

$Ag_3[Fe(CN)_6]$ was prepared from an aqueous solutions of $Ag(NO_3)_2$ (Sigma-Aldrich) and $K_3Fe(CN)_6$ (Sigma-Aldrich) mixed at room temperature with stirring as described previously.[S1] The resulting precipitate was filtered, washed with H_2O (3 $\times$ 5 ml) and dried at 50 $^\circ$C overnight to yield a brick-red polycrystalline solid.

Conversion of $Ag_3[Fe(CN)_6]$ to $KAg_3[Fe(CN)_6]$ was performed using a solution of potassium naphthalenide in 25 ml tetrahydrofuran (THF) under nitrogen. Stoichiometric potassium naphthalenide — prepared by the reaction, under nitrogen with at least 5 hours stirring, of potassium metal with naphthalene dissolved in 25 ml THF — was chilled to between $-5\,^\circ$C and 0 $^\circ$C with a salt-ice bath and added to $Ag_3Fe(CN)_6$. This mixture was stirred at between -5 to 0 $^\circ$C overnight. The resulting suspension was filtered then washed twice with 20 ml dry THF. The resulting brown powder was dried under vacuum for 2 hours.

Characterisation

Room temperature powder diffraction patterns were collected using a PANalytical X'Pert Pro diffractometer according to a 6 h scan regime using variable divergence slit width (fixed sample illumination of 10 cm) on a rotating sample.
Infrared spectra were collected at room temperature using a Bruker Tensor27 FT-IR spectrometer fitted with a diamond attenuated total reflection (ATR) module operated with the OPUS data collection programme.

2 Data & Refinements

Powder X-ray diffraction and infrared spectroscopy

Rietveld analysis of powder X-ray diffraction patterns was carried out with TOPAS Academic V4.1 by non-linear least squares refinement.[S2] In both cases variable divergence intensity with a TCHZ peak-shape and simple axial model were used. Special positions and constraints are noted in Tables S1 and S2 as appropriate.

Table S1: X-ray powder diffraction structural and refinement parameters for $Ag_3[Fe(CN)_6]$. Estimated standard deviations are given in parentheses. Atom positions were fixed to those determined in a previous neutron diffraction study.[S1]

R_{wp}	5.462			
a (Å)	7.0279(5)			
c (Å)	7.2546(5)			
V (Å^3)	310.31(5)			
Atom	x	y	z	B_{eq} (Å^2)
Fe†	0	0	0	5.36(14)
Ag†	$\frac{1}{2}$	0	$\frac{1}{2}$	8.50(6)
C‡	0.22037	0	0.16111	6.1(3)
N‡	0.33609	0	0.26732	6.1(3)

†Refined with anisotropic displacement parameters

‡Refined with isotropic displacement parameters constrained to be identical.

Table S2: X-ray powder diffraction structural and refinement parameters for $KAg_3[Fe(CN)_6]$ resulting from a two-phase refinement with $Ag_3[Fe(CN)_6]$ (see Table S1). Estimated standard deviations are given in parentheses. $Ag_3[Fe(CN)_6]$ structure is fixed to the values resulting from refinement of $Ag_3[Fe(CN)_6]$ alone. Atomic positions are fixed to those reported for $KMn[Ag(CN)_2]_3$ and were not refined.[S5] Weight percent of product may be underestimated due to peak anisotropy of $KAg_3[Fe(CN)_6]$.

R_{wp}	5.591				
Weight percent	85.7(4)				
a (Å)	7.0698(2)				
c (Å)	7.1836(3)				
V (Å^3)	310.95(3)				
Atom	x	y	z	Occ.	B_{eq} (Å^2)
K*	$\frac{1}{3}$	$\frac{2}{3}$	0	0.920(13)	4.1(4)
Fe	0	0	0	1	4.15(13)
Ag†	0.50467	0.00934	$\frac{1}{2}$	1	7.25(7)
C‡	0.2553	-0.0045	0.1669	1	8.1(4)
N‡	0.3462	0.0033	0.2837	1	8.1(4)

†Refined with anisotropic displacement parameters

‡Refined with isotropic displacement parameters constrained to be identical.

Figure S1: Infrared (IR) spectra are in excellent agreement with previous work on thin films of $Ag_3Fe(CN)_6$ and $KAg_3Fe(CN)_6$ made by electrochemical reduction. The corresponding asymmetric cyanide stretching frequencies reported in Ref. S3 are 2169 and 2093 cm^{-1}, respectively; here we observe these stretches at 2169.6 cm^{-1} and 2091.1 cm^{-1}. The π-bonding interaction between low-lying CN π^* and Fe t_{2g} orbitals is dependent on d-orbital occupancy. Reduction from M^{III} to M^{II} results in a red shift in the asymmetric C–N stretching due to greater availability of t_{2g} d-electrons, and therefore more significant back-bonding. There is a small residual reagent peak in the $KAg_3Fe(CN)_6$ pattern at 2169.7 cm^{-1}, consistent with the remaining starting material indicated by PXRD. The extra peaks in the starting material at 2113.898 and 2096.41 cm^{-1} are likely due to decomposition of the starting material. (Silver hexacyanoferrate changes colour from brick red to green over a period of months. IR spectra were collected over a month after synthesis, leaving sufficient time for some degradation.) All other IR active fundamental modes are below 600 cm^{-1}.

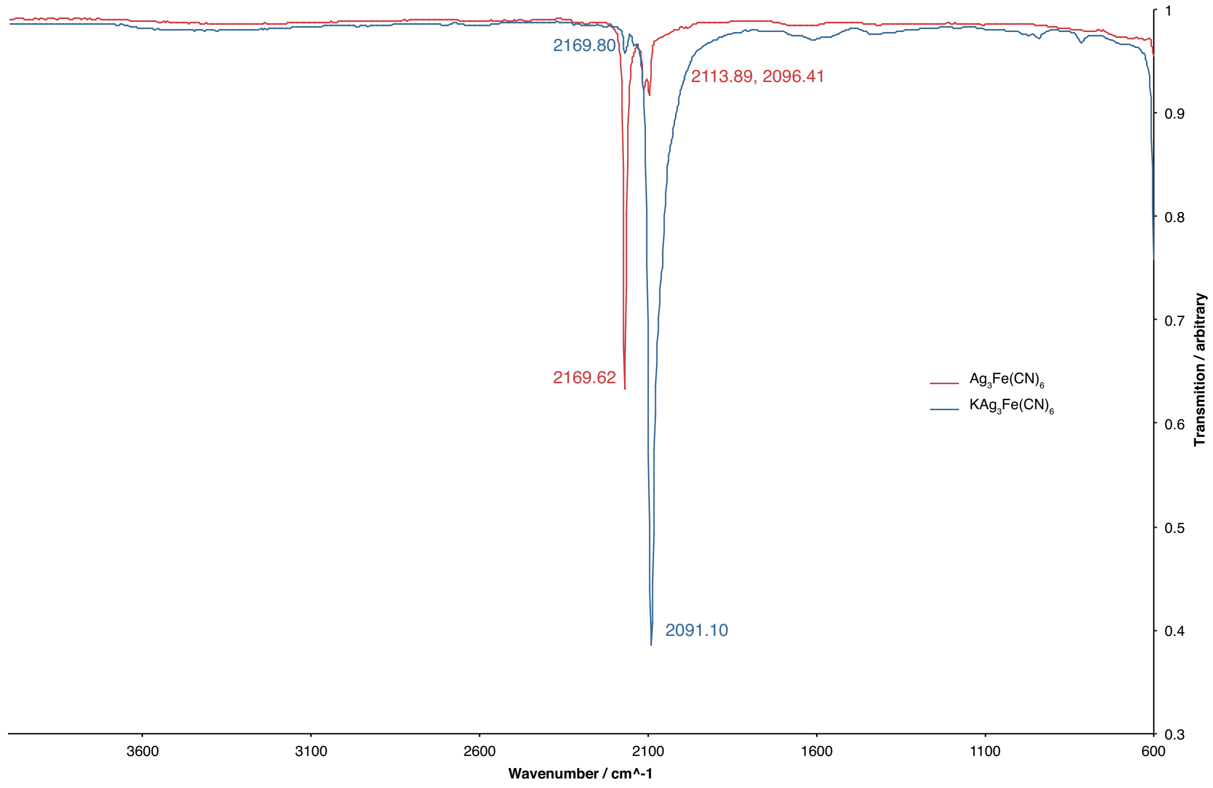

Table S3: Crystallographic data for various topical and canonical intercalation ion-storage compounds in deintercalated and intercalated states. Cell symmetry and size normalised to cell contents where necessary (indicated by $*$). In each case the intercalated ion is indicated in bold for the intercalated compound.

Compound	V (Å^3)	a (Å)	b (Å)	c (Å)	α (°)	β (°)	γ (°)	$\lvert\Delta V\rvert$ (Å^3)	$\lvert\Delta V/V\rvert$ (%)	Formula units per cell	e$^-$ per formula unit	Normalised $\lvert\Delta V\rvert$ (Å^3/ e$^-$)	Stored charge density (e$^-$/Å^3)	Ref.
FeSiO$_4$	341.45	10.3434	6.5779	5.0185	90	90	90	12.064	3.533	8	2.00	0.754	0.04686	S6
**Mg**FeSiO$_4$	329.38	10.2464	6.5038	4.9427	90	90	90							
Li$_{0.68}$CoO$_2$	97.31	2.8107	2.8107	14.2235	90	90	120	0.823	0.845	3	0.32	0.857	0.00987	S7
**Li**CoO$_2$	96.49	2.8156	2.8156	14.0542	90	90	120							
FePO$_4$	271.94	9.8195	5.79174	4.78166	90	90	90	19.198	7.060	4	1.00	4.799	0.01471	S8
**Li**FePO$_4$	291.14	10.3248	6.00715	4.69409	90	90	90							
Li$_{1.33}$Ti$_{1.67}$O$_4$	584.17	8.3595	8.3595	8.3595	90	90	90	1.194	0.204	8	1.00	0.149	0.01369	S9
**Li**$_{2.33}$Ti$_{1.67}$O$_4$	582.98	8.3538	8.3538	8.3538	90	90	90							
2,6-Napth(COOLi)$_2$	468.08	10.27	5.34	8.63	90	98.83	90	1.545	0.330	2	2.00	0.386	0.00855	S10
**Li**$_2$[2,6-Napth(COOLi)$_2$]	469.62	9.709	5.965	8.417	90	105.55	90							
C*	52.90	4.26	4.26	3.36	90	90	120	1.121	2.119	1	1.00	1.121	0.01890	S11
**Li**C$_6$	51.78	4.0307	4.0307	3.68	90	90	120							
Mn$_2$(CN)$_6$ *	1227.10	10.71	10.71	10.71	90	90	90	16.669	1.358	4	3.00	1.389	0.00978	S12
**Na**$_3$Mn$_2$(CN)$_6$ *	1243.77	10.459	10.905	10.905	90	90	90							
Na$_{0.5}$VP$_{4.8}$F$_{0.7}$	842.24	8.80	8.80	10.88	90	90	90	25.829	3.067	8	1.00	3.229	0.00950	S13
**Na**$_{1.5}$VP$_{4.8}$F$_{0.7}$	868.07	9.035	9.035	10.634	90	90	90							
NaFePO$_4$F	851.85	5.22	13.85	11.78	90	90	90	31.909	3.746	8	1.00	3.989	0.00939	S14
**Na**$_2$FePO$_4$F	819.94	5.1047	14.133	11.366	90	90	90							
Na$_{0.04}$Ni[Fe(CN)$_6$]$_{0.71}$	1058.09	10.19	10.19	10.19	90	90	90	6.242	0.590	4	0.80	1.951	0.00302	S15
**Na**$_{0.84}$Ni[Fe(CN)$_6$]$_{0.71}$	1064.33	10.21	10.21	10.21	90	90	90							
β-NaFeP$_2$O$_7$	562.09	6.33	9.46	11.10	62.611	83.29	72.242	18.962	3.374	3	1.00	6.321	0.00534	S16
**Na**$_2$FeP$_2$O$_7$	581.05	6.4490	9.4825	10.9933	64.847	86.240	73.133							
NaV$_2$(PO$_4$)$_3$	1321.57	8.43	8.43	21.49	90	90	120	120.909	9.149	6	2.00	10.076	0.00908	S17, S18
**Na**$_3$V$_2$(PO$_4$)$_3$	1442.48	8.738	8.738	21.815	90	90	120							
NaMn$_2$(CN)$_6$ *	1235.13	10.69	10.75	10.75	90	90	90	33.981	2.751	4	1.00	8.495	0.00324	S12
**Na**$_2$Mn$_2$(CN)$_6$ *	1201.15	10.658	10.616	10.616	90	90	90							
CuHCF	1015.08	10.05	10.05	10.05	90	90	90	27.516	2.711	4	0.72	9.554	0.00284	S19
**K**$_{0.7}$CuHCF	1042.59	10.14	10.14	10.14	90	90	90							
Ag$_3$Fe(CN)$_6$	310.31	7.03	7.03	7.25	90	90	120	0.640	0.206	1	0.92	0.696	0.00296	This work
**K**$_{0.92}$Ag$_3$Fe(CN)$_6$	310.95	7.0698	7.0698	7.1836	90	90	120							
C*	566.09	4.93	8.54	13.46	90	90	90	329.915	58.280	8	1.00	41.239	0.01413	S20
**K**C$_8$	896.00	4.92	8.51	21.4	90	90	90							

3 References

(S1) A. L. Goodwin, D. A. Keen, M. G. Tucker, M. T. Dove, L. Peters, and J. S. Evans. *J. Am. Chem. Soc.*, 130:9660–9661, 2008.

(S2) A. A. Coelho. *TOPAS-Academic, V4.1*, 2007. Coelho Software.

(S3) S. B. Moon, A. Xidis, and V. D. Neff. *J. Phys. Chem.*, 97(8):1634–1638, 1993.

(S4) L. H. Jones. *J. Chem. Phys.*, 26(6):1578–1584, 1957.

(S5) A. B. Cairns, A. L. Thompson, M. G. Tucker, J. Haines, and A. L. Goodwin. *J. Am. Chem. Soc.*, 2012.

(S6) Y. Orikasa, T. Masese, Y. Koyama, T. Mori, M. Hattori, K. Yamamoto, T. Okado, Z.-D. Huang, T. Minato, C. Tassel, J. Kim, Y. Kobayashi, T. Abe, H. Kageyama, and Y. Uchimoto. *Sci. Rep.*, 4:5622, 07 2014.

(S7) Y. Takahashi, N. Kijima, K. Dokko, M. Nishizawa, I. Uchida, and J. Akimoto. *J. Solid State Chem.* , 180(1):313–321, 2007.

(S8) A. Yamada, H. Koizumi, N. Sonoyama, and R. Kanno. *Electrochem. Solid St.*, 8(8):A409–A413, 2005.

(S9) S. Scharner, W. Weppner, and P. Schmid-Beurmann. *J. Electrochem. Soc.*, 146(3):857–861, 1999.

(S10) N. Ogihara, T. Yasuda, Y. Kishida, T. Ohsuna, K. Miyamoto, and N. Ohba. *Angew. Chem. Int. Ed.*, 53(43):11467–11472, 2014.

(S11) X.-L. Wang, K. An, L. Cai, Z. Feng, S. E. Nagler, C. Daniel, K. J. Rhodes, A. D. Stoica, H. D. Skorpenske, C. Liang, W. Zhang, J. Kim, Y. Qi, and S. J. Harris. *Sci. Rep.*, 2:747, 10 2012.

(S12) H.-W. Lee, R. Y. Wang, M. Pasta, S. Woo Lee, N. Liu, and Y. Cui. *Nature Commun.*, 5, doi:10.1038/ncomms6280 2014.

(S13) Y.-U. Park, D.-H. Seo, H.-S. Kwon, B. Kim, J. Kim, H. Kim, I. Kim, H.-I. Yoo, and K. Kang. *J. Am. Chem. Soc.*, 135(37):13870–13878, 2013.

(S14) B. L. Ellis, W. R. M. Makahnouk, Y. Makimura, K. Toghill, and L. F. Nazar. *Nature Mater.*, 6(10):749–753, 10 2007.

(S15) Y. You, X.-L. Wu, Y.-X. Yin, and Y.-G. Guo. *J. Mater. Chem. A*, 1:14061–14065, 2013.

(S16) P. Barpanda, G. Liu, C. D. Ling, M. Tamaru, M. Avdeev, S.-C. Chung, Y. Yamada, and A. Yamada. *Chem. Mater.*, 25(17):3480–3487, 2013.

(S17) Z. Jian, L. Zhao, H. Pan, Y.-S. Hu, H. Li, W. Chen, and L. Chen. *Electrochem. Commun.*, 14(1):86–89, 2012.

(S18) Z. Jian, W. Han, X. Lu, H. Yang, Y.-S. Hu, J. Zhou, Z. Zhou, J. Li, W. Chen, D. Chen, and L. Chen. *Adv. Energy Mater.*, 3(2):156–160, 2013.

(S19) C. D. Wessells, R. A. Huggins, and Y. Cui. *Nature Commun.*, 2:500, 2011.

(S20) P. Lagrange, D. Guerard, and A. Herold. *Ann. Chim.*, 3:143–159, 1978.
